# Supplementary material for: Isolation and Characterization of a Lytic Vibrio parahaemolyticus Phage vB_VpaP_GHSM17 from Sewage Samples
Source: Viruses. 2022 Jul 22;14(8):1601. doi: 10.3390/v14081601 (PMC9331696; doi:10.3390/v14081601)

Table S1. ORFs function analysis of phage vB_VpaP_GHSM17.

Coding DNA Sequence	Start	Stop	Len. (bp)	Annotation	Functional Group
CDS-1	307	663	357	hypothetical protein	
CDS-2	656	1069	414	peptidase M15A	Lysis
CDS-3	1078	1632	555	hypothetical protein	
CDS-4	1632	2246	615	bacterial Ig-like domain family protein	Additional functions
CDS-5	2255	2554	300	hypothetical protein	
CDS-6	2659	4578	1,920	terminase large subunit	DNA packaging
CDS-7	4589	4888	300	DNA maturase A	DNA metabolism
CDS-8	4898	7630	2,733	glycosyl hydrolase	Lysis
CDS-9	7639	8250	612	tail fibers protein	Structure
CDS-10	8271	12125	3,855	internal core protein	Structure
CDS-11	12176	13303	1,128	hypothetical protein	
CDS-12	13288	14931	1,644	internal virion protein	Structure
CDS-13	14941	15690	750	hypothetical protein	
CDS-14	15700	18045	2,346	tail tubular protein B	Structure
CDS-15	18055	18615	561	tail tubular protein A	Structure
CDS-16	18681	18809	129	hypothetical protein	
CDS-17	18821	19819	999	major capsid protein	Structure
CDS-18	19885	20700	816	scaffolding protein	Structure
CDS-19	20700	22232	1,533	head-tail connector protein	Structure
CDS-20	22241	22486	246	hypothetical protein	
CDS-21	22473	22892	420	GNAT family N-acetyltransferase	Additional functions
CDS-22	22924	25374	2,451	RNA polymerase	DNA metabolism
CDS-23	25563	26147	585	ATP-binding protein	Additional functions
CDS-24	26144	26263	120	hypothetical protein	
CDS-25	26263	26703	441	Endonuclease	DNA metabolism
CDS-26	26690	26896	207	hypothetical protein	
CDS-27	26883	27833	951	Exonuclease	DNA metabolism
CDS-28	27836	28261	426	hypothetical protein	
CDS-29	28271	28480	210	hypothetical protein	
CDS-30	28480	29109	630	Pyrophosphatase	Additional functions
CDS-31	29121	29453	333	hypothetical protein	
CDS-32	29499	30314	816	hypothetical protein	
CDS-33	30516	31112	597	hypothetical protein	
CDS-34	31121	31696	576	hypothetical protein	
CDS-35	31712	34138	2427	DNA polymerase	DNA metabolism
CDS-36	34131	34364	234	DNA helicase	DNA metabolism
CDS-37	34364	35644	1,281	putative DNA helicase	DNA metabolism

CDS-38	35626	36438	813	putative primase	DNA metabolism
CDS-39	36628	37731	1,104	hypothetical protein	
CDS-40	37757	39898	2,142	hypothetical protein	
CDS-41	39928	40344	417	hypothetical protein	
CDS-42	40331	40588	258	hypothetical protein	
CDS-43	40590	40739	150	hypothetical protein	
CDS-44	40748	41044	297	hypothetical protein	
CDS-45	41044	41580	537	hypothetical protein	

Table S2. The results of the genome sequence alignment of phage vB_VpaP_GHSM17.

Scientific Name	Family	Genus	Score	Q.C. (%)	E.	P.I. (%)	A.L. (bp)	Accession
Vibrio phage vB_VpaP_KF2	*Autographiviridae*	*Maculvirus*	67,774	99	0	94.82	43,571	NC_048036.1
Vibrio phage vB_VpP_NS8	*Autographiviridae*	*Maculvirus*	65,826	98	0	94.32	43,252	MZ592921.1
Vibrio phage BUCT233	*Autographiviridae*	*Maculvirus*	66,417	99	0	94.12	43,245	MZ020222.1
Vibrio phage vB_Vc_SrVc2	*Autographiviridae*	*Maculvirus*	61,792	97	0	93.92	43,157	MW331544.1
Vibrio phage vB_Vc_SrVc9	*Autographiviridae*	*Maculvirus*	61,765	97	0	93.91	43,156	LR794124.1
Vibrio phage vB_VpP_DE10	*Autographiviridae*	*Maculvirus*	64,670	97	0	93.37	42,871	MZ516827.1
Vibrio phage vB_VpP_FE11	*Autographiviridae*	*Maculvirus*	65,334	99	0	93.33	43,397	MT178448.1
Vibrio phage vB_VpP_DE17	*Autographiviridae*	*Maculvirus*	65,383	99	0	93.31	43,394	MW250641.1
Vibrio phage vB_VpaP_MGD1	*Autographiviridae*	*Maculvirus*	64,239	98	0	92.44	43,290	MT501516.1
Vibrio phage VP93	*Autographiviridae*	*Maculvirus*	61,397	97	0	92.37	43,931	FJ896200.1
Vibrio phage vB_VpaP_KF1	*Autographiviridae*	*Maculvirus*	67,538	99	0	92.23	43,237	NC_048035.1
Vibrio phage OWB	*Autographiviridae*	*Maculvirus*	56,579	98	0	88.57	43,264	MN974282.1
Vibrio phage vB_VpaS_OWB	*Autographiviridae*	*Maculvirus*	56,579	98	0	88.57	43,264	NC_048167.1

Table S3. All *Vibrio* phages in family *Autographiviridae.*

Scientific Name	Family	Genus/ Subfamily	Len. (bp)	Accession
Vibrio phage vB_VhaP_VH-5	*Autographiviridae*	*Beijerinckvirinae*	42,485	MN497414.1
Vibrio phage Vc1	*Autographiviridae*	*Colwellvirinae*	44,541	KJ502657.1
Vibrio phage 15E36.1	*Autographiviridae*	*Colwellvirinae*	45,211	MW865291.1
Vibrio phage phi-A318	*Autographiviridae*	*Colwellvirinae*	42,544	KF322026.1
Vibrio phage AS51	*Autographiviridae*	*Colwellvirinae*	42,542	KF800937.1
Vibrio phage Vp670	*Autographiviridae*	*Colwellvirinae*	43,121	KY290756.1
Vibrio phage vB_VpaP_MGD2	*Autographiviridae*	*Colwellvirinae*	45,105	MK820013.2
Vibrio phage VP-HS15	*Autographiviridae*	*Colwellvirinae*	46,759	MN807240.1
Vibrio phage VEN	*Autographiviridae*	*Colwellvirinae*	44,603	MG545917.1
Vibrio phage VP505	*Autographiviridae*	*Colwellvirinae*	43,009	MW451247.1
Vibrio phage 1.204.O._10N.222.46.F12	*Autographiviridae*	*Cyclitvirus*	44,168	MG592574.1
Vibrio phage BUCT233	*Autographiviridae*	*Maculvirus*	43,245	MZ020222.1
Vibrio phage OWB	*Autographiviridae*	*Maculvirus*	43,264	MN974282.1
Vibrio phage vB_Vc_SrVc2	*Autographiviridae*	*Maculvirus*	43,157	MW331544.1
Vibrio phage vB_Vc_SrVc9	*Autographiviridae*	*Maculvirus*	43,156	LR794124.1
Vibrio phage vB_VpaP_GHSM17	*Autographiviridae*	*Maculvirus*	43,228	OM362522
Vibrio phage vB_VpaP_KF1	*Autographiviridae*	*Maculvirus*	43,237	NC_048035.1
Vibrio phage vB_VpaP_KF2	*Autographiviridae*	*Maculvirus*	43,571	NC_048036.1
Vibrio phage vB_VpaP_MGD1	*Autographiviridae*	*Maculvirus*	43,290	MT501516.1
Vibrio phage vB_VpaS_OWB	*Autographiviridae*	*Maculvirus*	43,264	NC_048167.1
Vibrio phage vB_VpP_DE10	*Autographiviridae*	*Maculvirus*	42,871	MZ516827.1
Vibrio phage vB_VpP_DE17	*Autographiviridae*	*Maculvirus*	43,394	MW250641.1
Vibrio phage vB_VpP_FE11	*Autographiviridae*	*Maculvirus*	43,397	MT178448.1
Vibrio phage vB_VpP_NS8	*Autographiviridae*	*Maculvirus*	43,252	MZ592921.1
Vibrio phage VP93	*Autographiviridae*	*Maculvirus*	43,931	FJ896200.1
Vibrio phage vB_VpP_G1	*Autographiviridae*	*Melnykvirinae*	43,859	MZ592920.1
Vibrio phage ICP3	*Autographiviridae*	*Studiervirinae*	39,162	HQ641340.1
Vibrio phage N4	*Autographiviridae*	*Studiervirinae*	38,497	FJ409640.1
Vibrio phage VP4	*Autographiviridae*	*Studiervirinae*	39,503	DQ029335.1
Vibrio phage ICP3_2007_A	*Autographiviridae*	*Studiervirinae*	39,088	HQ641344.1
Vibrio phage ICP3_2008_A	*Autographiviridae*	*Studiervirinae*	39,349	HQ641343.1
Vibrio phage ICP3_2009_A	*Autographiviridae*	*Studiervirinae*	38,983	HQ641342.1
Vibrio phage ICP3_2009_B	*Autographiviridae*	*Studiervirinae*	39,042	HQ641341.1
Vibrio phage JSF11	*Autographiviridae*	*Studiervirinae*	39,341	KY883641.1
Vibrio phage JSF18	*Autographiviridae*	*Studiervirinae*	38,570	KY883650.1
Vibrio phage JSF20	*Autographiviridae*	*Studiervirinae*	39,378	KY883651.1
Vibrio phage JSF24	*Autographiviridae*	*Studiervirinae*	39,378	KY883652.1
Vibrio phage JSF25	*Autographiviridae*	*Studiervirinae*	38,593	MF574151.1

Vibrio phage JSF28	*Autographiviridae*	*Studiervirinae*	38,489	KY883643.1
Vibrio phage JSF30	*Autographiviridae*	*Studiervirinae*	38,056	KY883644.1
Vibrio phage JSF31	*Autographiviridae*	*Studiervirinae*	38,581	KY883645.1
Vibrio phage JSF32	*Autographiviridae*	*Studiervirinae*	38,581	KY883646.1
Vibrio phage JSF34	*Autographiviridae*	*Studiervirinae*	39,378	KY883653.1
Vibrio phage JSF35	*Autographiviridae*	*Studiervirinae*	38,688	KY883648.1
Vibrio phage JSF36	*Autographiviridae*	*Studiervirinae*	38,581	KY883649.1
Vibrio phage JSF37	*Autographiviridae*	*Studiervirinae*	38,451	MT215167.1
Vibrio phage Rostov 13	*Autographiviridae*	*Studiervirinae*	36,326	OK169295.1
Vibrio phage Rostov-1	*Autographiviridae*	*Studiervirinae*	37,247	MG957431.1
Vibrio phage VP3	*Autographiviridae*	*Studiervirinae*	39,481	JQ780163.1
Vibrio phage JSF7	*Autographiviridae*	*Tawavirus*	46,318	KY065149.1
Vibrio phage phiV141	*Autographiviridae*	*Tawavirus*	43,313	MT227925.1

Figure S1. Lysogenic validation of phage vB_VpaP_GHSM17.

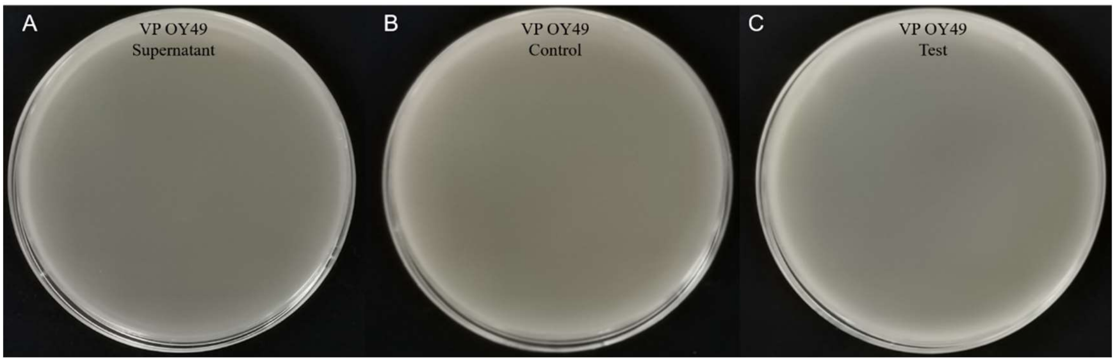

Supplement: Supplementary file 1 [file viruses-14-01601-s001.zip › viruses-1718931-supplementary.pdf]
